# The Landmark Series: Systemic Therapy for Resectable Gastrointestinal Stromal Tumors

**DOI:** 10.1245/s10434-020-08869-w

**Published:** 2020-07-30

**Authors:** Emily Z. Keung, Chandrajit P. Raut, Piotr Rutkowski

**Affiliations:** 1 The University of Texas MD Anderson Cancer Center, Houston, TX USA; 2 Brigham and Women’s Hospital/Dana-Farber Cancer Institute, Harvard Medical School, Boston, MA USA; 3 Maria Sklodowska-Curie National Research Institute of Oncology, Warsaw, Poland

## Abstract

Gastrointestinal stromal tumors (GISTs) are the most common mesenchymal neoplasms of the gastrointestinal tract. Complete resection is the only potentially curative treatment, although recurrence is common, occurring in approximately 40–50% of patients. The introduction of effective molecularly targeted therapies for GISTs has dramatically changed the clinical management paradigms for, and prognosis of, patients with intermediate- and high-risk GISTs, as well as those with locally advanced and metastatic disease. In this article, we review landmark studies that evaluated the use and efficacy of the tyrosine kinase inhibitors imatinib and sunitinib in the adjuvant and neoadjuvant settings for resectable primary and limited resectable metastatic GISTs.

Gastrointestinal stromal tumors (GISTs) are the most common mesenchymal tumors of the gastrointestinal tract, developing most commonly in the stomach and small intestine as a result of activating mutations in *KIT* or *PDGFRA*, genes encoding receptor protein tyrosine kinases. Over the past 2 decades, remarkable advances have been made in our understanding of GISTs and the development of molecular-targeted therapies, and tyrosine kinase inhibitors (TKIs) such as imatinib and sunitinib have dramatically changed the management and prognosis of patients with this malignancy. Although surgery is the treatment of choice and the only curative treatment for resectable GISTs, recurrence is common, particularly in patients with intermediate- and high-risk GISTs as defined by Miettinen and Lasota.^1^ On the heels of landmark clinical trials demonstrating remarkable response of imatinib and sunitinib in patients with advanced unresectable and metastatic GIST,^2^^–^4 there arose great interest in evaluating the safety and efficacy of using TKI therapy in the adjuvant and neoadjuvant settings for patients with resectable intermediate- and high-risk, locally advanced, or limited resectable metastatic disease. The landmark studies evaluating the use of imatinib for perioperative therapy in resectable GISTs are reviewed below.

## Adjuvant Therapy

The success of imatinib in the treatment of advanced unresectable and metastatic GIST^2^^–^4 led to great interest in using the drug in the adjuvant setting following primary tumor resection to prevent or delay recurrence and prolong survival. The role of imatinib in the adjuvant setting has been evaluated in several phase II and III clinical trials, as summarized below (Table 1).

**Table 1:** Landmark studies of adjuvant therapy for *KIT*-positive GISTs

Year published	Study name or reporting institution	Type of study	Eligibility criteria and cohort numbers	Major findings		Impact on practice
				Primary endpoints	Secondary endpoints	
2013	ACOSOG Z9000	Single-arm, multicenter, phase II	Adjuvant imatinib (400 mg/day) for 1 year Cohort (*n* = 106): Patients who underwent complete gross tumor removal, with high-risk tumor defined as ≥ 10 cm, intraperitoneal tumor rupture, or up to four peritoneal implants	1-year OS: 99% 3-year OS: 97% 5-year OS: 83%	1-year RFS: 96% 3-year RFS: 60% 5-year RFS: 40% 83% completed 1 year of adjuvant imatinib Tumor mutation status associated with RFS	1. Adjuvant imatinib was well tolerated and safe in the adjuvant setting 2. Confirmed impact of tumor mutation on the efficacy of adjuvant imatinib and RFS
2009, 2014	ACOSOG Z9001	Randomized, placebo-controlled, phase III	Adjuvant imatinib (400 mg/day) for 1 year Cohort (imatinib *n* = 359, placebo *n* = 354): Patients who underwent complete gross resection of GIST ≥ 3 cm	*Imatinib* 1-year RFS: 98% *Placebo* 1-year RFS: 83%		1. Adjuvant imatinib was well tolerated and safe in the adjuvant setting 2. Adjuvant imatinib was associated with improved RFS compared with placebo 3. US FDA approval of imatinib for postoperative treatment of adult patients after resection of *KIT*-positive GIST
2012, 2014, 2016, 2017, 2020	SSG XVIII AIO	Randomized, open-label, phase III	Adjuvant imatinib (400 mg/day) for 1 vs. 3 years Cohort (1 year of adjuvant imatinib, *n* = 200; 3 years of adjuvant imatinib, *n* = 200): Patients who underwent complete gross resection of GIST with tumor diameter > 10 cm, mitotic count > 10/50 HPFs, tumor diameter > 5 cm and mitotic count > 5/50 HPFs, or tumor rupture before or at the time of surgical resection	*1* *year of adjuvant imatinib* 5-year RFS: 52.3% 10-year RFS: 42% *3* *years of adjuvant imatinib* 5-year RFS: 71.1% 10-year RFS: 53%	*1* *year of adjuvant imatinib* 5-year OS: 85.3% 10-year OS: 65% *3* *years of adjuvant imatinib* 5-year OS: 91.9% 10-year OS: 79%	1. Established new standard of adjuvant imatinib therapy for 3 years for patients following complete resection of high-risk GISTs 2. Few patients developed recurrence while receiving adjuvant imatinib, suggesting that adjuvant therapy does not increase the risk of secondary resistance and that recurrence that develops after cessation of adjuvant imatinib may respond to rechallenge with imatinib
2015	EORTC 62024	Randomized, open-label, phase III	Adjuvant imatinib (400 mg/day) for 2 years vs. observation Cohort (imatinib *n* = 417, observation *n* = 454): Patients who underwent complete gross resection of high-risk GIST (tumor > 10 cm, mitotic count > 10/50 HPFs, or tumor > 5 cm and mitotic rate > 5/50 HPFs), or intermediate-risk GIST (tumor ≤ 5 cm and mitotic rate 6/50 to 10/50 HPFs or tumor size > 5 cm to 10 cm and mitotic rate ≤ 5/50 HPFs)	*2* *years of adjuvant imatinib* 5-year IFFS: 87% 5-year IFFS, high-risk: 89% *Observation* 5-year IFFS: 84% 5-year IFFS, high-risk: 73%	*2* *years of adjuvant imatinib* 3-year RFS: 84% 5-year RFS: 69% 5-year OS: 100% *Observation* 3-year RFS: 66% 5-year RFS: 63% 5-year OS: 99%	1. Supports use of adjuvant imatinib following complete resection of intermediate- and high-risk GISTs 2. Few patients developed recurrence while receiving adjuvant imatinib, suggesting that adjuvant therapy does not increase the risk of secondary resistance and that recurrence that develops after cessation of adjuvant imatinib may respond to rechallenge with imatinib
2018	PERSIST-5	Single-arm, prospective, phase II	Adjuvant imatinib (400 mg/day) for 5 years or until progression or intolerance Cohort (*n* = 91): Primary GIST at any site ≥ 2 cm with ≥ 5 mitoses/50 HPFs or non-gastric primary GIST ≥ 5 cm	5-year RFS: 90% No patient with imatinib-sensitive mutation recurred while receiving imatinib therapy	5-year OS: 95%	5 years of adjuvant therapy appeared safe and effective at controlling the recurrence rate

*ACOSOG* American College of Surgeons Oncology Group, *EORTC* European Organization for Research and Treatment of Cancer, *GISTs* gastrointestinal stromal tumors, *HPFs* high-power fields, *IFFS* imatinib failure-free survival, *OS* overall survival, *RFS* recurrence-free survival

## ACOSOG Z9000

### Purpose and Study Design

The American College of Surgeons Oncology Group (ACOSOG) performed the first trials of imatinib in the adjuvant setting. ACOSOG Z9000^5^ was a multicenter, single-arm, phase II study that enrolled 106 patients between September 2001 and September 2003, from 48 institutions, who underwent macroscopically complete resection of high-risk KIT-positive GISTs, defined as tumors ≥ 10 cm, those with intraperitoneal tumor rupture, or those with up to four peritoneal implants. Patients received adjuvant imatinib (400 mg/day) for 1 year and were imaged by computed tomography (CT) or magnetic resonance imaging every 5 months for the first 2 years then every 6 months for the following 3 years. The primary endpoint was to compare overall survival (OS) with that of historical controls, which was estimated to be 35% with surgery alone.

### Results

Overall, in this single-arm study, adjuvant imatinib was well tolerated and safe, with 83% of patients completing the prescribed year of therapy and 69% receiving the prescribed dose of 400 mg/day. The results of this study showed that postoperative imatinib for 1 year prolonged recurrence-free survival (RFS) after complete GIST resection and was also associated with improved OS compared with historic controls (3-year OS 97%, 5-year OS 83% vs. historic 5-year OS 35%). As expected, tumor mitotic rate, tumor location, and mutation status were associated with RFS on univariate analyses.

### Implications for Practice

ACOSOG Z9000/Z9001 showed that adjuvant imatinib for 1 year following macroscopically complete resection of primary GISTs was safe and well tolerated and improved RFS compared with placebo. Based on the results of ACOSOG Z9001, in December 2008 the US FDA approved imatinib for the postoperative treatment of adult patients after resection of KIT-positive GISTs. However, as many patients in both Z9000 and Z9001 recurred upon completion of therapy, the results of these studies suggested that adjuvant imatinib delays rather than prevents disease recurrence and that longer duration of adjuvant therapy may further improve RFS.

## ACOSOG Z9001

### Purpose and Study Design

**Fig. 1:**
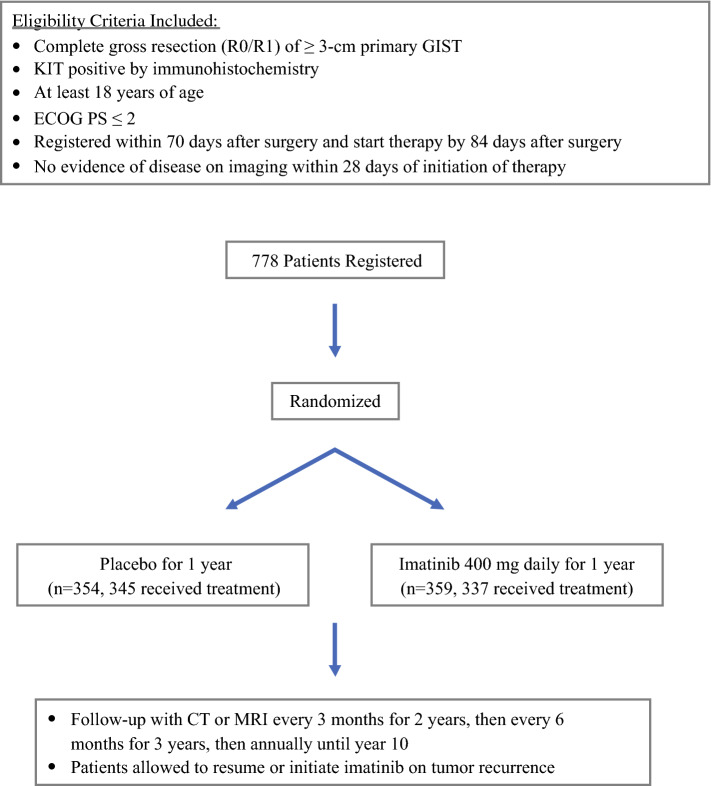
ACOSOG Z9001 study schema. *ACOSOG* American College of Surgeons Oncology Group, *GIST* gastrointestinal stromal tumor, *ECOG* Eastern Cooperative Oncology Group, *CT* computed tomography, *MRI* magnetic resonance imaging

ACOSOG Z9001^6^^,^^7^ was a randomized, double-blind, placebo-controlled, phase III trial of adjuvant imatinib for 1 year in patients who underwent complete gross resection of primary *KIT*-positive GISTs ≥ 3 cm (Fig. 1). Patients from 230 institutions were randomized to receive either adjuvant imatinib (*n* = 359) or placebo (*n* = 354); crossover was allowed in the event of tumor recurrence. Although the original primary endpoint was OS, 6 months prior to the first planned efficacy interim analysis the primary endpoint was changed to RFS when it became clear that the event (death) rate would be significantly lower than the event rate specified in the original statistical design because of the efficacy of imatinib and the crossover trial design that allowed patients who progressed on placebo to receive imatinib.

### Results

Median follow-up was 19.7 months (0–56.4), during which 30 (8%) patients in the imatinib group and 70 (20%) in the placebo group developed tumor recurrence or died. Adjuvant imatinib was associated with significantly improved RFS compared with placebo (98% vs. 83%) at 1 year and was well tolerated. No difference in OS was noted; however, this was not unexpected given the crossover design. Of note, patients who recurred generally did so shortly after completion of 1 year of adjuvant therapy. The slope of the RFS curve for the patients in the imatinib arm was similar to that of the placebo arm, but offset by approximately 18 months. Additionally, Z9001 enrolled many patients considered to be at low risk of recurrence, as subsequently defined by Miettinen and Lasota, as the risk categories were not well understood at the start of the trial.^1^

## Scandinavian/German SSG XVIII/AIO

### Purpose and Study Design

**Fig. 2:**
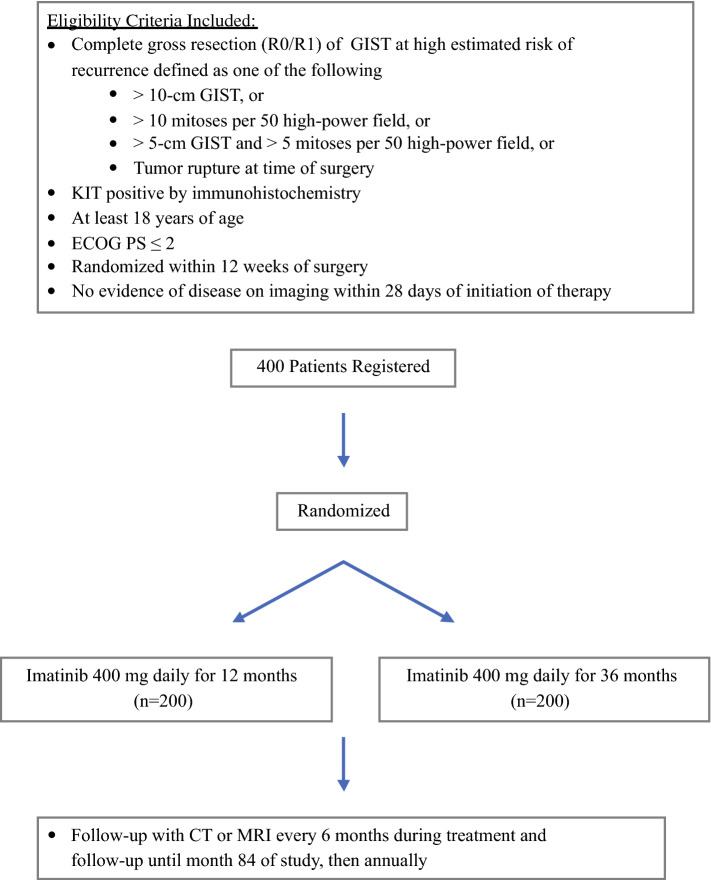
SSG XVIII/AIO study schema. *GIST* gastrointestinal stromal tumor, *ECOG PS* Eastern Cooperative Oncology Group performance score, *CT* computed tomography, *MRI* magnetic resonance imaging

The Scandinavian/German SSG XVIII/AIO trial^8^^–^12 was a randomized, open-label trial of 1 versus 3 years of postoperative imatinib (400 mg/day) after complete gross resection of high-risk *KIT*-positive GISTs following complete macroscopic resection (Fig. 2). High-risk GIST was defined as tumors with at least one of the following: tumor diameter > 10 cm, mitotic count > 10/50 high-power fields (HPF), tumor diameter > 5 cm and mitotic count > 5/50 HPFs, or tumor rupture before or at the time of surgical resection. A total of 400 patients from 24 hospitals were enrolled between 4 February 2004 and 29 September 2008.

### Results

Results after 10 years of follow-up were recently presented.^12^ Median follow-up was 119 months. Compared with patients randomized to receive 1 year of adjuvant imatinib, those randomized to receive 3 years of adjuvant imatinib were observed to have improved 5-year/10-year RFS (71/53% vs. 53/42%; hazard ratio [HR] 0.66, 95% confidence interval [CI] 0.49–0.87; *p* = 0.003) and 5-year/10-year OS (92/79% vs. 86/65%; HR 0.55, 95% CI 0.37–0.83; *p* = 0.04).

### Implications for Practice

This study established a new standard for treating patients after resection of high-risk GISTs with adjuvant imatinib for 3 years. The long-term data confirmed sustained OS benefit in the 3-year adjuvant imatinib arm. Of note, this study did include patients with GISTs now understood to be less sensitive to imatinib at 400 mg/day dosing (*KIT* exon 9 mutation), as well as those likely to be primarily resistant to imatinib (wild-type, *PDGFRA* D842V), with similar numbers in each trial arm.

## EORTC 62024^13^

### Purpose and Study Design

**Fig. 3:**
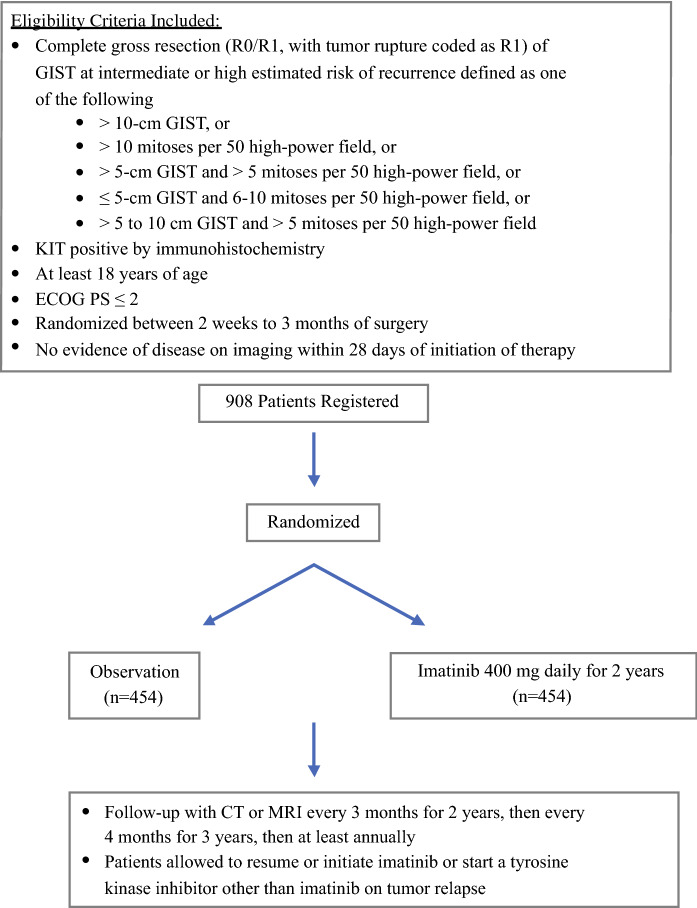
EORTC 62024 study schema. *EORTC* European Organization for Research and Treatment of Cancer, *GIST* gastrointestinal stromal tumor, *ECOG PS* Eastern Cooperative Oncology Group performance score, *CT* computed tomography, *MRI* magnetic resonance imaging

The European Organization for Research and Treatment of Cancer (EORTC) conducted the randomized, open-label, phase III EORTC 62024 trial^13^ to compare 2-year adjuvant treatment with imatinib versus observation alone in patients with intermediate- and high-risk primary KIT-positive GISTs (Fig. 3). The primary endpoint of this study was originally OS but was modified in 2009 to imatinib failure-free survival (IFFS), defined as time to death or starting a TKI other than imatinib, as an endpoint sensitive to secondary resistance to imatinib. Of 908 patients who were enrolled from 112 hospitals between December 2004 and October 2008, 835 patients were eligible, with 418 randomized to the observation arm and 417 randomized to the adjuvant imatinib arm.

### Results

Median follow-up was 56.4 months. There was no significant difference in IFFS between study arms, with 402 patients remaining imatinib failure-free in the imatinib arm and 391 patients remaining imatinib failure-free in the observation arm (3-year IFFS 90.8% vs. 90.8%, 5-year IFFS 87.0% vs. 84.1%; HR 0.79, 98.5% CI 0.5–1.25; *p* = 0.21). RFS was significantly better among patients who received adjuvant imatinib (3-year RFS 84.3% vs. 65.8%, 5-year RFS 69.4% vs. 62.9%; *p* < 0.01). OS did not differ between arms (3-year OS 96.8% vs. 96.3%, 5-year OS 91.8% vs. 92.7%).

### Implications for Practice

This study again supported the role of adjuvant imatinib in decreasing recurrence risk for patients with intermediate- and high-risk GISTs following complete macroscopic resection. However, the broader implication is that there is no difference in time to the start of second-line therapy whether imatinib is administered as an adjuvant therapy immediately after surgery or after recurrence.

## Persist-5^14^

### Purpose and Study Design

As 3 years of adjuvant imatinib therapy was shown to be associated with reduced recurrence rates and improved OS in patients with high-risk primary GISTs compared with those who received 1 year of therapy, the question arose as to whether a longer duration of adjuvant imatinib beyond 3 years might improve outcomes further. PERSIST-5 is a prospective, multicenter, single-arm, phase II study to evaluate whether adjuvant treatment with imatinib (400 mg/day) for 5 years is tolerable and efficacious. Eligible patients included those with a primary GIST of any location ≥ 2 cm with ≥ 5 mitoses/50 HPFs, or primary non-gastric GIST ≥ 5 cm. Ninety-one patients from 21 institutions were enrolled, with data collected from 5 August 2009 through 20 December 2016. Primary and secondary endpoints included 5-year RFS and OS, respectively.

### Results

Twenty-four (26%) patients had GISTs that were of intermediate risk of recurrence based on the Miettinen and Lasota classification,^1^ while 67 (74%) were of high risk. Median duration of adjuvant imatinib treatment was 55.1 months (range 0.5–60.6), with 46 patients (51%) completing 5 years of therapy. Forty-five patients (49%) discontinued treatment before 5 years because of patient choice (21%), adverse events (16%), or other reasons (12%). No patient with imatinib-sensitive mutations had disease recurrence while receiving therapy. Of the seven patients who recurred, one recurred while receiving imatinib but had *PDGFRA* D842V mutation, and six recurred after discontinuation of imatinib therapy.

### Implications for Practice

Adjuvant therapy with imatinib for 5 years was demonstrated to be safe and effective at controlling recurrence rates in patients with imatinib-sensitive mutations. However, compliance with this adjuvant regimen was challenging, with 49% of patients discontinuing therapy early. The results of the study emphasized the importance of pretreatment assessment of GIST molecular status as a driver of long-term benefit with adjuvant targeted therapy. It is unknown whether this longer 5-year duration of adjuvant imatinib therapy is associated with improved RFS and OS compared with 3-year adjuvant therapy, although the results appear comparable with those of EORTC 62024 and SSG XVIII/AIO. The results of this single-arm exploratory study is the basis of a randomized trial of 3-year versus 5-year adjuvant imatinib therapy (NCT 02413736).

## Neoadjuvant Treatment

The remarkable responses to imatinib in many patients with advanced unresectable and metastatic GISTs prompted the question of whether a neoadjuvant imatinib treatment approach might benefit patients in select clinical circumstances. Neoadjuvant therapy is an attractive treatment strategy to downstage disease, allow definitive resection, and improve local disease control in patients with locally advanced and/or marginally resectable solid tumors across histologies for whom upfront surgery may be technically challenging, overly morbid, or not feasible. Current European (European Society for Medical Oncology [ESMO]) and US (National Comprehensive Cancer Network [NCCN]) guidelines^15^^,^^16^ recommend consideration of preoperative imatinib and tumor mutational testing if surgical morbidity could be reduced by downstaging the tumor preoperatively. This approach is particularly attractive and indicated in cases when surgery may be technically challenging (rectum, duodenum, gastroesophageal junction) and tumor downstaging may facilitate tumor resectability (converting from an open laparotomy to a minimally invasive approach) or enable a less extensive/organ-sparing surgery, and may improve surgical outcomes such as likelihood of achieving a negative margin of resection and decreasing the risk of tumor perforation/rupture. Preoperative therapy with imatinib should be used until the maximum response is obtained (usually 6–12 months from the beginning of treatment) and before the development of secondary resistance to therapy. Response to therapy should be carefully monitored by imaging studies. As accurate assessment of recurrence risk cannot be made in patients who received preoperative systemic therapy, adjuvant imatinib should be used for at least 3 years following surgical resection. These recommendations are based on a limited number of landmark phase II and collaborative retrospective studies described below (Table 2).

**Table 2:** Landmark studies of neoadjuvant therapy for *KIT*-positive GISTs

Year published	Study name or reporting institution	Type of study	Eligibility criteria and cohort numbers	Major findings			Year published
				Primary endpoints	Secondary endpoints	Impact on practice	
2008, 2012	RTOG 0132/ACRIN 6665	Multi-institution, non-randomized, phase II	Neoadjuvant imatinib (600 mg/day) for 8–12 weeks followed by resection in patients with SD/PR and adjuvant imatinib for 2 years Cohort A (*n* = 31): Primary GIST ≥ 5 cm Cohort B (*n* = 22): Potentially resectable metastatic/recurrent GIST ≥ 2 cm	*Cohort A* 2-year OS: 93.5% 2-year DSS: 93.5% 5-year OS: 76.9% 5-year DSS: 76.9% *Cohort B* 2-year OS: 90.9% 2-year DSS: 100% 5-year OS: 68.2% 5-year DSS: 77.3%	*Cohort A* 2-year PFS: 83.9% 5-year PFS: 56.7% *Cohort B* 2-year PFS: 77.3% 5-year PFS: 29.8%	Grade 3: 34% Grade 4: 21% Grade 5: 2%	1. First prospective study of preoperative imatinib in GISTs 2. Preoperative imatinib therapy for 8–12 weeks was shown to be safe and well tolerated 3. Long-term follow-up showed a significant drop in PFS and OS after 2 years when adjuvant imatinib was discontinued
2009	MD Anderson Cancer Center	Single-institution, randomized, phase II	Neoadjuvant imatinib (600 mg/day) for 3 (*n* = 7), 5 (*n* = 6), or 7 (*n* = 6) days followed by resection and adjuvant imatinib for 2 years Cohort: Primary GIST ≥ 1 cm (*n* = 19)		1-year DFS: 94% 2-year DFS: 87%		1. Preoperative imatinib therapy was shown to be safe and well tolerated 2. Radiographic response to imatinib can be observed within the first week of therapy by ^18^FDG-PET and dCT, as well as histologically
2013	EORTC STBSG	Collaborative retrospective series of pooled databases from 10 centers	Neoadjuvant imatinib (400 mg/day) for a median of 40 weeks (range 6–190) Cohort: Locally advanced, non-metastatic GIST (*n* = 161)	5-year OS: 87% 5-year DSS: 95%	5-year DFS: 65%		1. Higher observed partial response rate compared with RTOG 0132/ACRIN 6665, likely related to the longer median duration of preoperative imatinib treatment, supporting recommendations to continue neoadjuvant therapy until maximal response was achieved prior to surgical resection

*ACRIN* American College of Radiology Imaging Network, *dCT* dynamic computed tomography, *DFS* disease-free survival, *DSS* disease-specific survival, *EORTC* European Organization for Research and Treatment of Cancer, ^*18*^*FDG-PET* ^18^fluorodeoxyglucose positron emission tomography, *GIST*s gastrointestinal stromal tumors, *OS* overall survival, *PFS* progression-free survival, *PR* partial response, *RTOG* Radiation Therapy Oncology Group, *SD* stable disease, *STBSG* Soft Tissue and Bone Sarcoma Group

## RTOG 0132/ACRIN 6665^17^^,^^18^

### Purpose and Study Design

The Radiation Therapy Oncology Group (RTOG) 01321 was a prospective, multicenter, non-randomized, phase II trial evaluating the efficacy and tolerability of preoperative imatinib in patients with *KIT*-positive resectable intermediate- to high-risk primary (≥ 5 cm) or recurrent/metastatic (≥ 2 cm) GISTs. Patients were enrolled from 18 RTOG institutions and were treated with imatinib (600 mg/day) for 8–12 weeks prior to surgery. Imatinib was stopped on the day prior to surgery and resumed as soon as possible postoperatively for 2 years. Clinical endpoints included assessments of imatinib-related toxicity, surgical complication assessment, GIST response to preoperative therapy, time to progression, progression-free survival (PFS), disease-specific survival, and OS.

### Results

Most primary GISTS (*n* = 30) presented in the stomach (52%), followed by the small bowel (20%), and median tumor size was 8.7 cm (range 5–24.5). Among patients with primary GISTs, stable disease (SD) and partial response (PR) were observed in 83% and 7% of patients, respectively, while in those with recurrent or metastatic disease (*n* = 22), SD and PR were observed in 91% and 4.5% of patients, respectively. Estimated 2-year OS and PFS were 93.5% and 83.9%, respectively, for patients with primary GIST, and 90.9% and 77.3%, respectively, for patients with recurrent or metastatic disease. Overall, neoadjuvant imatinib was well tolerated, with rates of grade 3, 4, and 5 postoperative toxicities of 34%, 20.8%, and 1.9%, respectively. The 8- to 12-week delay in the time to surgery did not appear to have any adverse effects on surgical outcomes.

### Implications for Practice

Initial results of RTOG 0132/American College of Radiology Imaging Network (ACRIN) 6665 were reported in 2008 and demonstrated that preoperative imatinib for 8–12 weeks was safe and well tolerated. Although survival outcomes at 2 years compared favorably with historical single-institution surgical series for patients with intermediate- to high-risk GISTs, it was unclear whether the apparent survival benefits seen in this study could be attributed to the use of imatinib preoperatively, or due to the 2 years of postoperative imatinib therapy. Follow-up results of this study were reported in 2012 and were notable for a significant drop in PFS and OS after 2 years when adjuvant imatinib was discontinued, supporting the need for longer durations of imatinib treatment in patients at intermediate- and high-risk of GIST recurrence following resection.

## MD Anderson, McAuliffe et al.^19^

### Purpose and Study Design

The group at The University of Texas MD Anderson Cancer Center (McAuliffe et al.^19^) performed a prospective, single-institution study to evaluate the safety and efficacy of preoperative imatinib (600 mg/day) in 19 patients with *KIT-*positive resectable GISTs (≥ 1 cm). Patients were randomized to receive imatinib for 3, 5, or 7 days preoperatively (*n* = 7, 6, and 6, respectively), with the last dose administered the morning of surgery. Preoperative tumor response to imatinib was assessed radiographically by dynamic CT (dCT) and ^18^fluorodeoxyglucose positron emission tomography (^18^FDG-PET), as well as histologically. Imatinib was resumed postoperatively for 2 years.

### Results

This study provided evidence that radiographic response to preoperative imatinib can be observed and assessed as early as within 1 week of initiation of therapy. Most patients in this study responded to preoperative imatinib as assessed by ^18^FDG-PET and dCT (69% and 71%, respectively). Similar to the results of RTOG 0132/ACRIN 6665, preoperative imatinib was well tolerated and safe. The observed 2-year disease-free survival (DFS) was 87%, with a median DFS of 46 months. Survival benefit of preoperative imatinib could not be determined as all patients received imatinib postoperatively for 2 years.

### Implications for Practice

Although a small study (*n* = 19), this single-institution, phase II trial demonstrated that preoperative imatinib was safe, and additionally showed that tumor metabolic response to TKI therapy occurs and can also be assessed within 1 week of treatment by ^18^FDG-PET.

## EORTC STBSG^20^

### Purpose and Study Design

The EORTC Soft Tissue and Bone Sarcoma Group (STBSG) performed a retrospective study to assess the long-term results of preoperative imatinib therapy in 161 consecutive patients with primary *KIT-*positive GISTs treated with preoperative imatinib between 2002 and 2011 at 10 sarcoma centers of the EORTC-STBSG. This remains the largest study to date of neoadjuvant imatinib therapy. Preoperative imatinib was continued until either maximal response, defined as two consecutive CT scans not showing further tumor regression, or if the surgeon deemed that resection was possible, whichever was achieved first.

### Results

Median follow-up was 46 months. Tumors were located in the stomach (55.3%), rectum (20.5%), duodenum (9.9%), ileum/jejunum (9.3%), esophagus (3.1%), and other locations (1.9%). The median time of preoperative imatinib therapy in this study was 40 weeks (range 6–190); 129 patients (80.1%) had documented response to preoperative therapy and 30 patients (18.6%) had documented stabilization of disease prior to surgery. The median duration of adjuvant imatinib therapy was 19 months (range 12–76). This study reported excellent safety data and long-term results, with a 5-year DFS and OS of 65% and 87%, respectively.

### Implications for Practice

The number of PRs was higher in this large retrospective series compared with that observed in the RTOG 0132-ACRIN 6665 trial, likely related to the longer duration of preoperative imatinib therapy and thus supporting the recommendation that preoperative imatinib therapy be continued until maximal tumor response is achieved. Survival benefit of preoperative imatinib could not be determined as all patients received imatinib postoperatively.

## Combination of Targeted Therapy and Metastasectomy

Cytoreductive surgery for recurrent or metastatic GISTs may be considered in select circumstances, including in patients presenting with oncologic emergencies such as hemorrhage, intestinal perforation, or obstruction, as well as in patients whose disease is stable or responsive to TKI therapy and when complete gross resection is possible and in patients with limited disease progression.^16^

In the pre-imatinib era, surgical resection of recurrent and metastatic GISTs was associated with improved survival if complete gross resection could be achieved.^21^ However, complete resection was often difficult to achieve due to the multifocal nature of recurrent and metastatic GISTs. In the post-imatinib era, TKI therapy is the standard first-line treatment for patients with metastatic, recurrent, and/or inoperable GISTs. Although maximal response to imatinib is typically achieved within 6–18 months of treatment,^22^ complete remissions are rare and median time to recurrence/progression on imatinib is within 2 years.^23^^,^^24^ Thus, cytoreductive surgery to resect residual disease became an attractive approach to investigate. Although prospective randomized trials did not prove feasible, a number of retrospective series of patients with recurrent and/or metastatic GISTs treated surgically after treatment with imatinib and/or sunitinib therapy have been published supporting the role of surgery for residual metastatic disease in patients with GISTs responding to imatinib (Table 3).^24^^–^42

**Table 3:** Landmark studies of metastasectomy for GISTs treated with tyrosine kinase inhibitor therapy

Authors, year published	Number of cases, clinical indications	Key results
Raut et al.^25^	*N* = 69 patients who underwent surgery for advanced GISTs while receiving TKI therapy Group I (*n* = 23): surgery at stable disease Group II (*n* = 32): surgery at limited progression Group III (*n* = 14): surgery at generalized progression	Group I: 1-year PFS 80%, 1-year OS 95% Group II: 1-year PFS 33%, 1-year OS 86% Group III: 1-year PFS 0%, 1-year OS 0%
Rutkowski et al.^42^	*N* = 141 patients who underwent surgery for initially inoperable and/or metastatic GISTs after receiving imatinib therapy Group I (*n* = 24): resection of residual disease after complete/partial response or lack of further response Group II (*n* = 8): surgery as salvage therapy for progression after initially successful imatinib therapy	Median follow-up 12 months Group I: Four recurrences of 5 patients who did not resume imatinib postoperatively, 1 recurrence of 19 patients who resumed imatinib postoperatively Group II: 5/8 patients progressed
Gronchi et al.^39^	*N* = 159 patients with advanced/metastatic GISTs treated initially with imatinib Group I (*n* = 27): surgery at response Group II (*n* = 8): surgery at progression	Group I: 1-year PFS 96%, 1-year OS 100%; 2-year PFS 69% Group II: 1-year PFS 0%, 1-year OS 60%
DeMatteo et al.^38^	*N* = 40 patients with metastatic GISTs treated with TKI therapy Group I (*n* = 20): surgery at response Group II (*n* = 13): surgery at focal progression Group III (*n* = 7): surgery at multifocal progression	Median follow-up 15 months Group I: 2-year PFS 61%, 2-year OS 100% Group II: 2-year PFS 24%, 2-year OS 36%, median TTP 12 months Group III: 1-year OS 36%, median TTP 3 months
Mussi et al.^37^	*N* = 80 patients with metastatic GISTs treated with TKI therapy Group I (*n* = 49): surgery at best response Group II (*n* = 31): surgery at focal progression	Morbidity in 13 patients Group I: 2-year PFS 64.4%, median PFS not reached, 5-year DSS 82.9%, median DSS not reached Group II: 2-year PFS 9.7%, median PFS 8 months, 5-year DSS 67.6%, median DSS not reached
Raut et al.^36^	*N* = 50 patients with metastatic imatinib-resistant GISTs undergoing surgery following sunitinib therapy Group I (*n* = 10): responsive disease Group II (*n* = 22): limited progression Group III (*n* = 18): generalized progression	Complication rate 54%, 48% R2 resection Median follow-up 15.2 months Median PFS and OS after surgery 5.8 and 16.4 months, respectively Median PFS and OS after the start of sunitinib 15.6 and 26 months, respectively Differences in PFS and OS between groups were not significant
Tielen et al.^35^	*N* = 55 patients with advanced/metastatic GISTs treated with TKI therapy Group I (*n* = 35): responders Group II (*n* = 20): nonresponders	Group I: 48% recurrence/progression, median PFS and OS not reached, 5-year OS 78% Group II: 85% recurrence/progression, median PFS 4 months, median OS 25 months, 3-year OS 26%
Bauer et al.^26^	*N* = 239 patients with GISTs undergoing surgery for metastatic GISTs Group I (*n* = 177): complete gross resection (R0/R1) Group II (*n* = 62): incomplete gross resection (R2)	Group I: median OS 8.7 years, median OS was not reached when surgery was performed at remission, median TTP was not reached Group II: median OS 5.3 years, median OS 5.1 years when surgery was performed at remission, median TTP 1.9 years when surgery was performed at response Groups I and II: no difference in median PFS in patients progressing at the time of surgery
Du et al.^34^	*N* = 41 of 210 planned patients with recurrent/metastatic GISTs treated with TKI therapy Arm A (*n* = 19): surgery Arm B (*n* = 22): imatinib alone	Group I: 2-year PFS 88.4%, median OS not reached Group II: 2-year PFS 57.7%, median OS 49 months
Fairweather et al.^33^	400 operations performed in 323 patients with metastatic GISTs treated with TKI therapy Group 1 (*n* = 64): surgery at response Group 2 (*n* = 100): surgery at stable disease Group 3 (*n* = 132): surgery at limited progression Group 4 (*n* = 104): surgery at generalized progression	In patients receiving imatinib before surgery, radiographic response was predictive of PFS and OS from the time of surgery Group 1: PFS 36 months, OS not reached Group 2: PFS 30 months, OS 144 months Group 3: PFS 11 months, OS 105 months Group 4: PFS 6 months, OS 66 months

Adapted from Rutkowski and Hompes^22^

*DSS* disease-specific survival, *GISTs* gastrointestinal stromal tumors, *OS* overall survival, *PFS* progression-free survival, *TKI* tyrosine kinase inhibitor, *TTP* time to progression

## ChiCTR-TRC-00000244^34^

### Purpose and Study Design

Two clinical trials (NCT00956072 in Europe, ChiCTR-TRC-00000244 in China) were attempted to address the question of whether patients with metastatic GISTs receiving imatinib would benefit further from surgical resection of residual disease. Both studies were closed early due to poor accrual, although ChiCTR-TRC-00000244 reported data on 41 of 210 planned patients. In ChiCTR-TRC-00000244, patients were treated with imatinib (400 mg/day) and those responding to therapy were randomized to either surgery for resection of residual disease with resumption of imatinib postoperatively (*n* = 19) or imatinib therapy alone (*n* = 22). All patients continued imatinib until disease progression

### Results

Patients in the surgery arm underwent surgery 3–12 months after initiating imatinib therapy, with 15 patients achieving PR and 7 patients achieving SD at the time of randomization. Of those in the imatinib-alone arm, 9 achieved PR and 10 achieved SD at the time of randomization. Two-year PFS was 88.4% in the surgery arm versus 57.7% in the imatinib-alone arm, with a median follow-up of 23 months (*p* = 0.089). Median OS was not reached in the surgery arm, and was 49 months in the imatinib-alone arm (*p* = 0.024).

### Implications for Practice

Although there was some suggestion of improved PFS with surgery compared with imatinib therapy alone, the difference was not statistically significant. It is possible that surgery may prolong survival in carefully selected patients, although this decision should be made at expert centers and following multidisciplinary discussion.

## Retrospective studies examining disease control after resection in selected patients with limited metastatic disease after IMATINIB treatment

Despite the lack of prospective, randomized controlled trials, the conclusions of multiple available retrospective series have overall been consistent. Although complete excision of residual metastatic lesions is associated with improved prognosis in these retrospective series,^24^^–^40 outcomes are consistently dependent on preoperative responses to imatinib, and it has not been prospectively demonstrated that the survival benefit associated with surgical resection of residual metastatic disease is due to surgery itself or to patient selection.

Currently available data do not support a clinical benefit of surgery for patients with generalized disease progression on TKI therapy.^25^^,^^38^^,^^42^ Raut et al. reported that 1-year PFS following surgical resection was 80%, 33% and 0% in patients with advanced GIST who achieved SD, limited progression, and generalized progression, respectively, with imatinib therapy preoperatively.^25^ Similarly, Rutkowski et al. found that patients who achieved PR or SD had significantly longer PFS and OS following surgical resection when compared with patients with progressive disease. The role of surgery in patients with advanced GISTs with focally progressive disease on imatinib, as well as those on lines of systemic therapy beyond imatinib, is limited^23^^,^^36^ and should be individualized and considered in an expert and multidisciplinary setting.

## Conclusion

The development of TKIs has dramatically altered the management landscape and improved outcomes of patients with GISTs. Initially limited to use in the metastatic setting, TKIs have since been shown to have utility both in the neoadjuvant and adjuvant settings. The rationale use of TKI therapy in the metastatic, neoadjuvant, and adjuvant settings requires knowledge of GIST mutational status, obtained through tumor biopsies prior to initiation of systemic therapy. Although imatinib and subsequent generations of TKIs have to date primarily benefitted patients with GISTs harboring common *KIT* mutations, avapritinib, the most recent TKI to receive FDA approval, demonstrates efficacy in patients with GISTs harboring the *PGDFR* D842V mutation. Future work will likely evaluate novel therapies, including avapritinib in combination with surgical management, for growing subsets of patients with GIST.
